# QiShenYiQi Pills, a compound in Chinese medicine, protects against pressure overload-induced cardiac hypertrophy through a multi-component and multi-target mode

**DOI:** 10.1038/srep11802

**Published:** 2015-07-02

**Authors:** Yuan-Yuan Chen, Quan Li, Chun-Shui Pan, Li Yan, Jing-Yu Fan, Ke He, Kai Sun, Yu-Ying Liu, Qing-Fang Chen, Yan Bai, Chuan-She Wang, Bing He, Ai-Ping Lv, Jing-Yan Han

**Affiliations:** 1 Department of Integration of Chinese and Western Medicine, School of Basic Medical Sciences, Peking University, Beijing, China; 2 Tasly Microcirculation Research Center, Peking University Health Science Center, Beijing, China; 3 Key Laboratory of Microcirculation, State Administration of Traditional Chinese Medicine of China, Beijing, China; 4 Key Laboratory of Stasis and Phlegm, State Administration of Traditional Chinese Medicine of China, Beijing, China; 5 Institute of Vascular Medicine, Peking University Third Hospital and Key Laboratory of Cardiovascular Molecular Biology and Regulatory Peptide, Ministry of Health, Beijing, China; 6 The School of Chinese Medicine of Hong Kong Baptist University, Kowloon Tong, Hong Kong, China

## Abstract

The present study aimed to explore the holistic mechanism for the antihypertrophic effect of a compound in Chinese medicine, QiShenYiQi Pills (QSYQ) and the contributions of its components to the effect in rats with cardiac hypertrophy (CH). After induction of CH by ascending aortic stenosis, rats were treated with QSYQ, each identified active ingredient (astragaloside IV, 3, 4-dihydroxy-phenyl lactic acid or notoginsenoside R1) from its 3 major herb components or dalbergia odorifera, either alone or combinations, for 1 month. QSYQ markedly attenuated CH, as evidenced by echocardiography, morphology and biochemistry. Proteomic analysis and western blot showed that the majority of differentially expressed proteins in the heart of QSYQ-treated rats were associated with energy metabolism or oxidative stress. Each ingredient alone or their combinations exhibited similar effects as QSYQ but to a lesser extent and differently with astragaloside IV and notoginsenoside R1 being more effective for enhancing energy metabolism, 3, 4-dihydroxy-phenyl lactic acid more effective for counteracting oxidative stress while dalbergia odorifera having little effect on the variables evaluated. In conclusion, QSYQ exerts a more potent antihypertrophic effect than any of its ingredients or their combinations, due to the interaction of its active components through a multi-component and multi-target mode.

Cardiac hypertrophy (CH) is initially an adaptive response to pressure or volume stress, which is characterized by increased cardiomyocyte size, re-expression of fetal genes, and activation of signaling pathways governing protein synthesis^1^. However, persistent and severe CH becomes a maladaptive response and ultimately contributes to subsequent heart failure^2^ that is a major and growing public health concern, as well as a leading cause of morbidity and mortality worldwide^3^. Thus, it is critical to suppress CH timely to prevent the progression of CH to heart failure.

CH is a multifactorial clinical syndrome induced by a diversity of stimuli such as pressure over-load, ischemic disease and genetic cardiac defects^4^, which manifests many pathological features including cardiac dysfunction, cardiac fibrosis, energy deficit, cardiomyocyte death, vascular dysfunction and oxidative stress^5^, involving inherently multiple and complex signaling pathways^6^. Currently, angiotensin-converting enzyme inhibitors, angiotensin receptor blockers, β-blockers and Ca^2+^ channel blockers are the major agents for management of CH in clinic^7^, while the mortality and morbidity of the syndrome remain unacceptably high^8^. This might be partly due to the fact that each of these agents acts on a single signaling pathway or target. We advocated that for complex multifactorial chronic diseases, treatment regimens that contain multiple drugs directing towards multi-pathways and multi-targets would possess stronger therapeutic efficacies^9^.

QiShenYiQi Pills (QSYQ) is a compound Chinese medicine approved by the State Food and Drug Administration of China in 2003 for treatment of cardiac dysfunction^10^, which is composed of Astragalus membranaceus (Huangqi), Salvia miltiorrhiza (Danshen), Panax notoginseng (Sanqi), and Dalbergia odorifera (Jiangxiang, DO). The major active ingredients are astragaloside IV (ASIV, from Huanqi), 3, 4-dihydroxy-phenyl lactic acid (DLA, from Danshen), and notoginsenoside R1 (R1, from Sanqi)^11^. Our previous study demonstrated that QSYQ could attenuate pressure over-load induced CH^12^. A recently published study showed that QSYQ exhibits a similar role as angiotensin II receptor antagonist valsartan in protection of cardiac hypertrophy in rats after 4 weeks treatment, and a more significant role than valsartan does after 8 weeks treatment, suggesting the advantage of QSYQ over the currently used cares in long-term treatment^13^. Other results revealed that ASIV has protective effect on CH induced by isoproterenol through attenuating inflammatory cytokines^14^. DLA can prevent isoproterenol-induced myocardial hypertrophy and improve cardiac function through acting as an antioxidant^15^. However, the underlying mechanism thereby QSYQ attenuates CH is poorly understood. Particularly, the contribution of each ingredient of QSYQ to its pharmacological activities is unknown.

In this study, using rat ascending aortic stenosis (AAS) model and proteomic and biochemical analyses, we investigated the holistic mechanisms underlying the therapeutic effect of QSYQ on CH. By comparing the efficacies and mechanisms of QSYQ, its single ingredient ASIV, DLA, R1, DO and various ingredient combinations we showed the rationality of QSYQ formula design, supporting that a regime containing multiple components is more effective than individual treatment for complex diseases^16^.

## Results

### The effect of different drug treatments on CH

**Figure 1:**
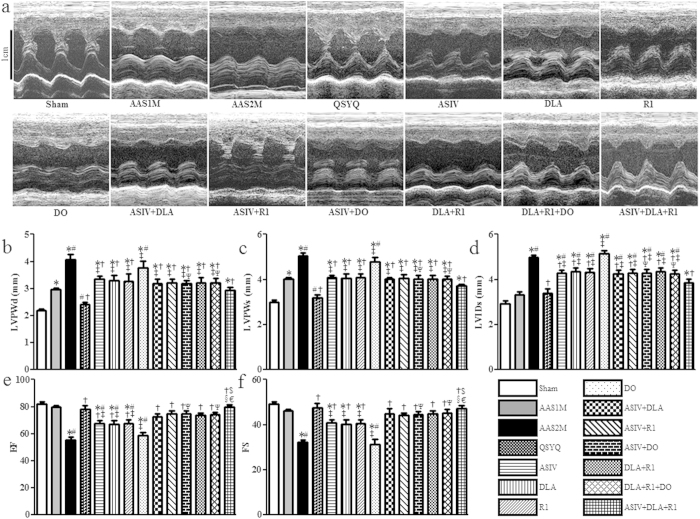
The effect of different drug treatments on echocardiographic parameters. **a** Representative rat echocardiograms in various conditions for determination of the ventricle wall thickness during cardiac cycles. **b-f** Quantitative measurement of LVPWd (**b**), LVPWs (**c**), LVIDs (**d**), EF (**e**), and FS (**f**). The data are presented as mean ± S.E. *p < 0.05 vs. Sham, ^#^p < 0.05 vs. AAS1M, ^†^p < 0.05 vs. AAS2M, ^‡^p < 0.05 vs. QSYQ, ^$^p < 0.05 vs. ASIV, ^§^p < 0.05 vs. DLA, ^€^p < 0.05 vs. R1, ^Ψ^p < 0.05 vs. DO. n = 6.

The representative images of M-mode echocardiograms from each group are presented in Fig. 1a. Note that compared to sham group, the heart in 1 month (30 days) of AAS group (AAS1M) exhibited marked hypertrophy as shown by a significantly increased left ventricular posterior wall thickness at end diastole and systole (LVPWd and LVPWs). These alterations became more prominent in 2 months (60 days) of AAS group (AAS2M) and accompanied with increased left ventricular internal diameter-systole (LVIDs) (Fig. 1a–d). Pressure over-load 2 months also led to cardiac dysfunction as suggested by reduction in left ventricle ejection fraction (EF) and fractional shortening (FS) (Fig. 1e,f). QSYQ treatment significantly restored LVPWd, LVPWs, LVIDs, EF and FS after AAS2M. The mono-therapies including ASIV, DLA, R1 and the combination therapies ASIV+DLA, ASIV+R1, ASIV+DO, DLA+R1, DLA+R1+DO, and ASIV+DLA+R1 also attenuated these impairments, but, except for ASIV+DLA+R1, were less effective in terms of the effect on LVPWd, LVPWs and LVIDs as compared with QSYQ. QSYQ and ASIV+DLA+R1 also showed stronger efficacy for restoring EF and FS than each related mono-therapy. Note that DO alone did not show any beneficial role in terms of any echocardiographic parameters.

**Figure 2:**
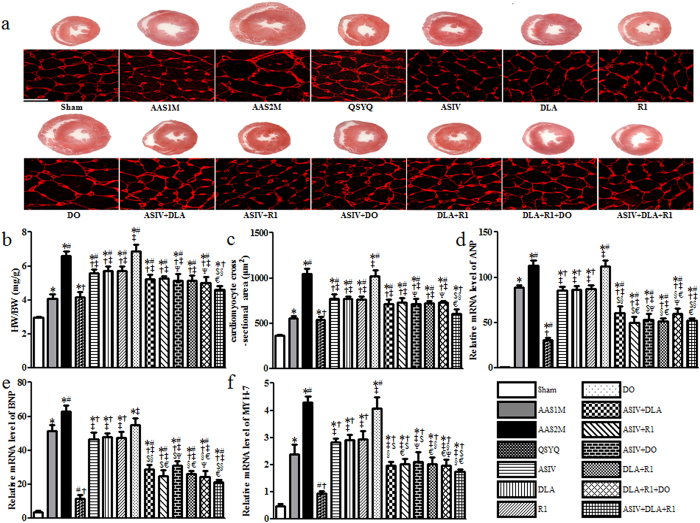
The effect of different drug treatments on CH. **a** Representative rat heart cross sections in different conditions with sections stained by WGA showing below demarcating cell boundaries (Bar = 25 μm). The heart sections were cut across the middle of heart vertical axis. **b** Quantitative evaluation of HW/BW. **c** Quantitative measurement of cardiomyocyte cross-sectional area. **d–f** Semi-quantitative analysis of ANP (**d**), BNP (**e**) and MYH-7 (**f**) mRNA level. The data are presented as mean ± S.E. ^*^p < 0.05 vs. Sham, ^#^p < 0.05 vs. AAS1M, ^†^p < 0.05 vs. AAS2M, ^‡^p < 0.05 vs. QSYQ, ^$^p < 0.05 vs. ASIV, ^§^p < 0.05 vs. DLA, ^€^p < 0.05 vs. R1, ^Ψ^p < 0.05 vs. DO. n = 6.

The results from echocardiography were confirmed by heart macro morphology and biochemistry studies. CH occurred obviously in AAS1M rats, and further exaggerated in AAS2M, as indicated by the increase in whole-heart cross section area and cardiomyocyte size (Fig. 2a,c), as well as in the ratio of heart weight (HW) to body weight (BW) (Fig. 2b). All drug treatments but DO significantly protected against these alterations compared to AAS2M, with QSYQ being the most effective one. Of interest, ASIV+DLA+R1 showed an effect comparable to QSYQ (Fig. 2a–c).

As CH markers, the expressions of atrial natriuretic peptide (ANP), brain natriuretic peptide (BNP) and β-myosin heavy chain (MYH-7) were determined in each group. As shown in Fig. 2d–f, the expressions of CH markers varied in different groups in a fashion similar to that in HW/BW, whole heart section area and cardiomyocyte size, suggesting that combination of two or more ingredients was superior to any single ingredient alone in treatment of CH.

### The effect of different drug treatments on histology of myocardium and myocardial blood flow (MBF)

**Figure 3:**
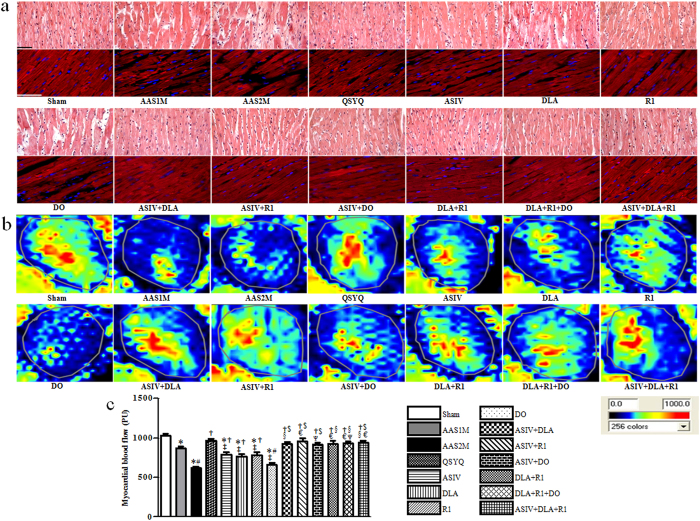
The effect of different drug treatments on myocardium histology and MBF. **a** Representative myocardium sections stained by HE showing cadiomyocyte hypertrophy (Bar = 50 μm) with sections stained for F-actin by rhodamine phalloidin showing below to illustrate cardiomyocyte rupture (Bar = 100  m) in different groups. **b** Representative images of MBF acquired by Laser Scanning Doppler Perfusion Imager in each group. **c** Statistical results of MBF in each group. The data are presented as mean ± S.E. ^*^p < 0.05 vs. Sham, ^#^p < 0.05 vs. AAS1M, ^†^p < 0.05 vs. AAS2M, ^‡^p < 0.05 vs. QSYQ, ^$^p < 0.05 vs. ASIV, ^§^p < 0.05 vs. DLA, ^€^p < 0.05 vs. R1, ^Ψ^p < 0.05 vs. DO. n = 6.

Rat myocardium from each group was examined by microscopy using hematoxylin and eosin (HE) and F-actin staining. As noticed in Fig. 3a, AAS led to tissue edema, myocardial fiber thickening and F-actin rupture, which were more prominent in AAS2M than in AAS1M. Clearly, these AAS-induced morphological alterations in myocardium were attenuated in the rats received all drug treatments except DO. Particularly, QSYQ and the combination treatments were more effective in relieving these alterations than any mono-therapy.

Figure 3b shows the color images acquired by the Laser Scanning Doppler and Fig. 3c is the quantitative evaluation of MBF in each group. An apparent decrease in MBF occurred in AAS1M, which further reduced in AAS2M. Consistent with the results from morphologic evaluation, all drug treatments except DO protected MBF from AAS-elicited decrease. Likewise, QSYQ and all the combination treatments exhibited a higher efficacy than any mono-therapy.

### Proteomic analysis of the differentially expressed proteins in each group

**Figure 4:**
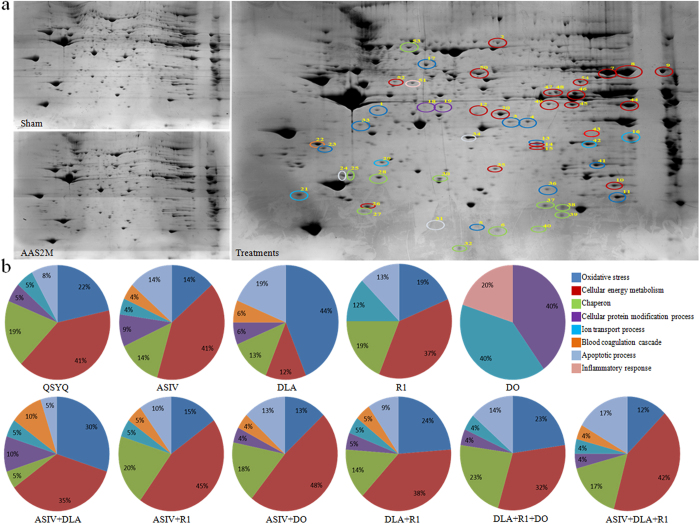
Proteomic analysis of the differentially expressed proteins in each group. **a** Representative images of the PhastGel Blue R stained two-dimensional gels of Sham, AAS2M and treatments group. Colorful circles in treatments indicate the differentially expressed spots from all drug treatments groups. **b** The proportion of various functional category in differentially expressed proteins in each group. n = 3.

To further gain insight into the mechanisms of different treatments for CH, proteomic analysis was undertaken by two-dimensional polyacrylamide gel electrophoresis for each group. Figure 4a shows the representative images of the PhastGel Blue R stained two-dimensional gels for Sham, AAS2M, and treatments group, respectively. Noticeably, the expression levels of numerous spots altered after AAS challenge for 2 months, which was protected by different drug treatments, as justified by Image Master 2D Platinum 7. Among all of the spots, 54 spots appeared to be significantly changed. The peptides were extracted from each differentially expressed spot using in-gel trypsin digestion, and were identified using mass spectrometry (MS). The results of the MS analysis of the 54 spots were summarized in Supplementary Table S1, showing that the 54 sports were identified as 41 proteins. The biological functions of these proteins can be sorted into eight groups: oxidative stress (PTGR2, ALDR, COQ7, SODM, PDIA3, COQ9, CRYM, GPX1, ETFA, and ESTD), energy metabolism (D3ZZN, ATPA, D4AOTO, NDUAA, ECH1, PEBP1, HPRT, ALDOA, ACOT2, ENOB, IDH3B, ODPA, ACADS, ALDH2, and GUAD), chaperon (HSPB6, HSPB1, HSPB2, HSPB7, CRYAB, and HSP70), protein modification (ARHL1), inflammatory response (FIBG), ion transport (VDAC1, VDAC2, TCTP and CLIC4), apoptosis (GDIRI, FRIH, and FHIL2), and blood coagulation (ANXA5). The differentially expressed spots were circled in the image of two-dimensional gel from treatments group using different colors based on the biological function classification (Fig. 4a).

To provide clue to understand the molecular mechanisms of each drug, we analyzed the functional distribution of these differentially expressed proteins in different groups, and the results are illustrated in Fig. 4b, wherein the proportion of each function regulated by a drug treatment is shown by the pie charts. In QSYQ, the proteins involved in energy metabolism and oxidative stress accounted for 41% and 22%, respectively, with the highest significance, indicating these two processes as the main mechanisms for the therapeutic function of QSYQ. The functional distribution of these differentially expressed proteins varied among different mono-therapies, with ASIV mainly affecting energy metabolism (41%), DLA mainly affecting oxidative stress process (44%), R1 affecting energy metabolism (37%) and DO affecting ion transport process (40%) or protein modification (40%). Interestingly, all combination treatments acted on the proteins implicated in energy metabolism and oxidative stress as their major targets.

The function of a protein depends on the biological context in which the protein acts, thus we identified canonical pathways affected by these differentially expressed proteins. A pattern analysis was performed using the reliable software MetaCore to analyze and build the biological pathways. The pathways regulated by differentially expressed proteins in QSYQ were found mainly related to energy metabolism and oxidative stress including: Glycolysis and gluconeogenesis (short map); Glycolysis and gluconeogenesis p.3; Glycolysis and gluconeogenesis p.3/Human version; Transcription_P53 signaling pathway; Oxidative stress Role of Sirtuin 1 and PGC1-alpha in activation of antioxidant defense system; Apoptosis and Survival inhibition of ROS-induced apoptosis by 17beta-erstradiol; Transcription Role of Akt in hypoxia induced HIF1 activation; G-protein signaling Regulation of RAC1 activity; G-protein signaling-Rac2 regulation pathway; G-protein signaling RhoA regulation pathway; G-protein signaling RhoB regulation pathway; Oxidative stress Angiotensin II-induced production of ROS. We selected the proteins involved in energy metabolism pathways (fructose-bisphosphate aldolase A (ALDOA), enolase α (ENOα), enolase β (ENOβ), delta(3,5)-delta(2,4)-dienoyl-CoA isomerase (ECH1), hypoxia-inducible factor 1 (HIF1), heat shock protein 70 (HSP70)) and oxidative stress pathways (Superoxide dismutase [Mn] (SODM), Rho GDP-dissociation inhibitor 1 (RhoGDI1) and heat shock protein B1 (HSPB1)) for further verification of holistic mechanisms of QSYQ on CH, all of which were identified by 2D as differentially expressed proteins except HIF1 which was selected based on pattern analysis.

### Assessment of energy metabolism

**Figure 5:**
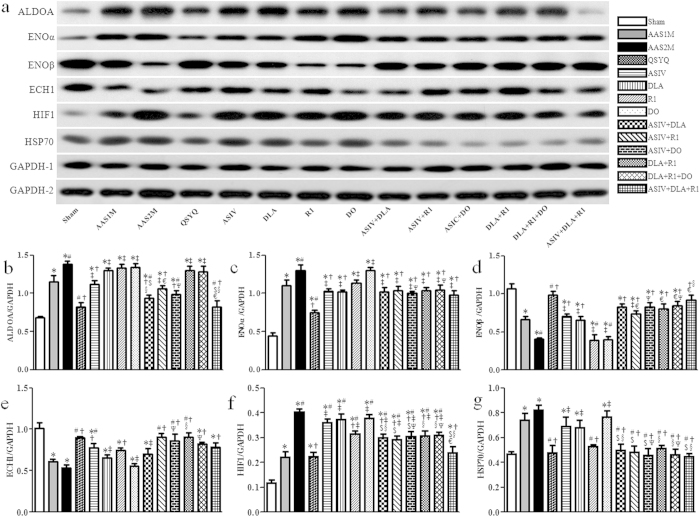
Western blot of proteins related to energy metabolism pathway in different groups. **a** The representative western blotting bands of each protein in different groups. **b–g** The semi-quantitative analysis of ALDOA (**b**), ENOα (**c**) ENOβ (**d**), ECH1 (**e**), HIF1 (**f**) and HSP70 (**g**) in various groups. The bands of ALDOA, ENOβ, HIF1 and GAPDH-2 were cropped from one gel, while the bands of ECH1, ENOα, HSP70 and GAPDH-1 were cropped from another gel, as shown in Supplementary Fig. S5a with indication of molecular size. The quantification was based on the data of 3 independent experiments and normalized to GAPDH. The data are presented as mean ± S.E. ^*^p < 0.05 vs. Sham, ^#^p < 0.05 vs. AAS1M, ^†^p < 0.05 vs. AAS2M, ^‡^p < 0.05 vs. QSYQ, ^$^p < 0.05 vs. ASIV, ^§^p < 0.05 vs. DLA, ^€^p < 0.05 vs. R1, ^Ψ^p < 0.05 vs. DO. n = 4.

The expressions of 6 enzymes (ALDOA, ENOα, ENOβ, ECH1, HIF1, HSP70) related to energy metabolism were assessed by western blot in different groups. As shown in Fig. 5, AAS challenge led to an increase in the expression of 4 enzymes, including ALDOA, ENOα, HIF1, and HSP70, while a decrease in the expression of ENOβ and ECH1, with AAS2M impairing these proteins more than AAS1M except ECH1 and HSP70. QSYQ restored all the AAS-induced alterations significantly. Mono and combination therapies affected the expression of energy metabolism-related proteins differently depending on the enzyme concerned. For example, ASIV mono-therapy and the ASIV containing combinations inhibited the elevation of ALDOA after AAS, which was intensified by combination with DLA or DLA+R1, while DLA, R1, DO alone and their combinations showed no effect on ALDOA (Fig. 5b). On the other hand, mono-therapy ASIV, DLA or R1 and all the combination therapies showed nearly equally beneficial role for ENOα (Fig. 5c). ASIV, DLA alone and all the combination therapies displayed a beneficial role, but R1 and DO had no effect, in the case of ENOβ (Fig. 5d). ASIV, R1 alone and all combinations except ASIV+DLA increased the expression of ECH1 (Fig. 5e). Interestingly, R1 alone and all the combination therapies showed significant inhibition on HIF1 and HSP70 (Fig. 5f,g). Of notice, in any case, DO alone did not show any effect while QSYQ was always among the most effective treatments.

**Figure 6:**
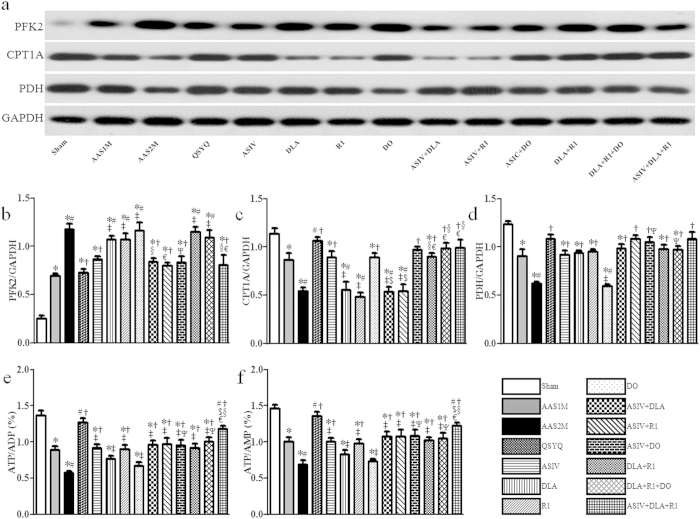
The effect of different drug treatments on enzymes related to energy metabolism tested by western blot and ELISA. **a–d** The representative western blotting bands and respected semi-quantitative analysis of PFK2 (**b**), CPT1A (**c**) and PDH (**d**), n = 4. **e–f** Quantitative measurement of ATP/ADP (**e**) and ATP/AMP (**f**) by ELISA in each group, n = 6. All bands in figure 6 were cropped from one gel, as shown in Supplementary Fig. S6a with indication of molecular size. The quantification was based on the data of 3 independent experiments and normalized to GAPDH. The data are presented as mean ± S.E. ^*^p < 0.05 vs. Sham, ^#^p < 0.05 vs. AAS1M, ^†^p < 0.05 vs. AAS2M, ^‡^p < 0.05 vs. QSYQ, ^$^p < 0.05 vs. ASIV, ^§^p < 0.05 vs. DLA, ^€^p < 0.05 vs. R1, ^Ψ^p < 0.05 vs. DO.

To further gain insight into the mechanism for the effect of different treatments on energy metabolism in CH, we determined the expression of 6-phosphofructo-2-kinase/fructose-2, 6-bisphosphatase (PFK2), Carnitine palm acyltransferase 1A (CPT1A) and pyruvate dehydrogenase (PDH) in different groups; the results are presented in Fig. 6. As a glycolysis regulator, PFK2 increased significantly in AAS groups, which was protected by QSYQ, ASIV mono- and ASIV containing combination treatments but not others (Fig. 6a,b). In contrast, CPT1A, an enzyme involved in fatty acid oxidation, significantly reduced in AAS groups compared to Sham, which was restored by all the treatments except DLA and R1 mono-therapy or their combination with ASIV (Fig. 6a,c). PDH, a key enzyme of glucose oxidation, significantly decreased in AAS groups as well compared to Sham. All drug treatments but DO restored the expression of PDH as compared to AAS2M group (Fig. 6a,d).

Moreover, adenosine triphosphate (ATP), adenosine diphosphate (ADP) and adenosine monophosphate (AMP) contents in cardiac tissue from different groups were assessed by enzyme-linked immuno sorbent assay (ELISA). As shown in Fig. 6e,f, both ATP/ADP and ATP/AMP were significantly down-regulated in AAS groups compared to Sham. Except DLA and DO alone, all the treatments protected this down-regulation significantly with QSYQ being most effective. Noticeably, QSYQ exhibited more effective than any other combination treatments except ASIV+DLA+R1, which showed more significant effect than its any composition.

### Assessment of oxidative stress

**Figure 7:**
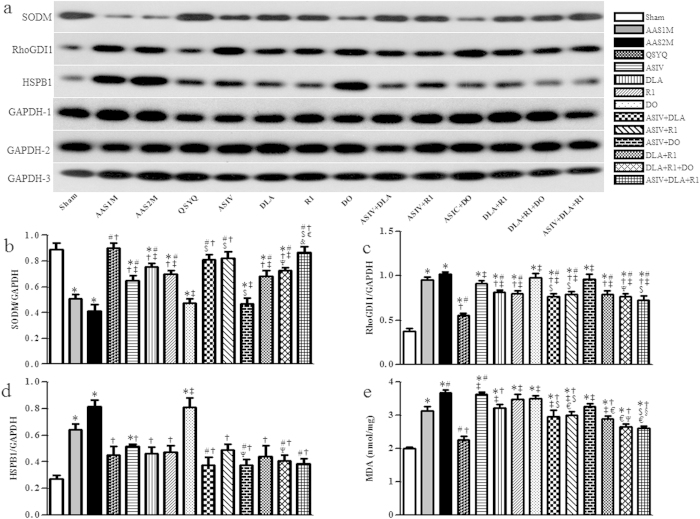
The effect of different drug treatments on oxidative stress tested by western blot and ELISA. **a–d** The representative western blotting bands and respective semi-quantitative analysis of SODM (**b**), RhoGDI1 (**c**) and HSPB1 (**d**), n = 4. **e** Quantitative evaluation by ELISA of MDA level in each group, n = 6. The bands of SODM and GAPDH-1 were cropped from one gel, the bands of RhoGDI1 and GAPDH-2 were cropped from one gel, and the bands of HSPB1 and GAPDH-3 were cropped from one gel, as shown in Supplementary Fig. S7a with indication of molecular size. The quantification was based on the data of 3 independent experiments and normalized to GAPDH. The data are presented as mean ± S.E. ^*^p < 0.05 vs. Sham, ^#^p < 0.05 vs. AAS1M, ^†^p < 0.05 vs. AAS2M, ^‡^p < 0.05 vs. QSYQ, ^$^p < 0.05 vs. ASIV, ^§^p < 0.05 vs. DLA, ^€^p < 0.05 vs. R1, ^Ψ^p < 0.05 vs. DO.

SODM, RhoGDI1 and HSPB1, the three proteins related to oxidative stress, were assessed by western blot. As a major endogenous antioxidant, SODM obviously reduced in AAS groups compared to Sham. This down-regulation was protected significantly by all the treatments except DO and ASIV+DO with QSYQ and ASIV+DLA+R1 being most effective (Fig. 7a,b). In contrast, the expression of RhoGDI1 increased significantly in AAS groups. Again, QSYQ was the most effective treatment for restoration of RhoGDI1. Interestingly, DLA, R1 and the combinations containing DLA and/or R1 relieved the expression of RhoGDI1, whereas ASIV, DO and ASIV+DO did not show any significant effect (Fig. 7a,c). AAS challenge remarkably increased HSPB1, and all treatments except DO alleviated the expression of HSPB1 (Fig. 7a,d). In addition, as shown in Fig. 7e, the content of methane dicarboxylic aldehyde (MDA) increased significantly in AAS1M compared to sham group, and further increased in AAS2M, while QSYQ, DLA and the combinations containing DLA attenuated MDA content. Of notice, ASIV and R1 alone did not show any effect on MDA, whereas ASIV+R1 obviously reduced the MDA level. Among these effective drug treatments, QSYQ again was the most effective one.

## Discussion

Consistent with our previous study^12^, the present study demonstrated the protective effect of QSYQ on pressure overload-induced CH in rats, as shown by improved heart function and macro and micromorphology. Similar to QSYQ, the active ingredients of QSYQ ASIV, DLA and R1 and their combinations also exerted an antihypertrophic effect but with a less potency. These results proved the necessity of including all the ingredients in QSYQ, and provided further evidence supporting QSYQ as a potential option for protection of CH.

QSYQ has been used in China for prevention and treatment of cardiac dysfunction^10^. However, our understanding of the mechanism for its beneficial role in CH and cardiac failure remains incomplete. The mechanisms so far reported accounting for the role of QSYQ in CH or cardiac failure include interference in inflammation^12^,^17^, oxidation and apoptosis^18^, but none of which has been accepted as a conclusive one. We speculate that as QSYQ is a compound medicine it will thus act via multiple mechanisms. To fully understand these mechanisms, a comprehensive strategy is needed instead of focusing only one or two aspects of the mechanisms. We thus undertook a proteomic analysis of cardiac tissues taken from different groups of rats and revealed a total of 54 spots (41 proteins) that altered significantly in the expression after drug treatments compared with AAS2M group. These proteins can be classified functionally into 8 groups, suggesting that QSYQ plays CH-protective role via a complex mechanism involving multiple processes, including those that have been reported respectively in different studies. More importantly, of the QSYQ-induced significantly altered proteins, 41% were related to energy metabolism and 22% to oxidative stress, indicating these two processes as the dominant mechanisms for QSYQ to exert effect on CH.

In agreement with the widely recognized fact that CH is accompanied by energy deficiency^19^, our results revealed a significant decrease in the ratio of ATP/ADP and ATP/AMP after AAS challenge. Interestingly, QSYQ inhibited this AAS-elicited reduction, demonstrating the ability of QSYQ to improve energy metabolism, a potential that has been found in other pathological conditions, such as cardiac ischemia-reperfusion injury and doxorubicin-induced myocardial structure damage and cardiac dysfunction, in which QSYQ increased myocardial ATP content via the up-regulation of ATP 5D^20^,^21^. The present study did not evaluate the effect of QSYQ on the expression of ATP synthase δ subunit, but rather assessed the changes in glycolysis and fatty acid β-oxidation in cardiac tissue. In consistence with the well-accepted view that CH is characterized by an increase in glycolysis and a decrease in fatty acid oxidation^22^, our results revealed that AAS challenge caused an increase in the proteins that are implicated in glycolysis, including ALDOA^23^, HIF1^24^, HSP70^25^, and PFK2^26^, but a decrease in the proteins that are related to oxidation of fatty acids and glucose, including ECH1^27^, CPT1A^28^ and PDH^29^,^30^. These results demonstrated a shift of metabolism to fetal profile in AAS-induced cardiac hypertrophy^22^,^31^, which was further verified by the fact that ENOα, the fetal form of ENO, increased, while ENOβ, the adult form of ENO^32^, decreased in AAS-challenged rats. Impressively, all these changes were protected by QSYQ treatment, suggesting that QSYQ improves energy metabolism in AAS-induced cardiac hypertrophy via modulation of metabolic profile. Nonetheless, whether QSYQ affects ATP synthase δ subunit in AAS-induced cardiac hypertrophy requires elucidation by further study.

Accumulating evidence demonstrates the critical role of oxidative stress in development of CH^33^. Previous studies showed that antioxidant SODM plays a role in preserving cardiac function by scavenging reactive oxygen species^34^,^35^. In addition, RhoGDI1 is known to play an important part in initiating oxidative stress, and inhibition of RhoGDI1 was reported to prevent angiotensin II-induced lipid peroxidation in myocardial hypertrophy^36^. Consistent with these results, in our study, MDA increased significantly after AAS for 2 months, indicating the presence of oxidative stress^37^, with a concurrency of increased RhoGDI1 and decreased SODM. QSYQ reversed these alterations, which confirms antioxidation as one of the mechanisms underlying CH-protective potential of QSYQ. The antioxidative potential of QSYQ was also indicated by a decrease in HSPB1 in QSYQ-treated rats. HSPB1 is known to be increased in CH in response to oxidative stress^38^.

It is predicable that improvements of energy metabolism and anti-oxidation are the dominant mechanisms for QSYQ to attenuate CH, since its major ingredients ASIV and R1 have been shown to improve energy deficiency^39^,^40^, while DLA antagonize oxidative stress^15^,^41^. In line with these results, our proteomic analysis results revealed that the largest portion of differential proteins in ASIV and R1 were related to energy metabolism. In addition, ASIV and R1, but not DLA and DO, increased ATP/ADP and ATP/AMP. Western blot showed that compared with DLA and DO, ASIV and R1 modulated more energy metabolism-related proteins with ASIV affecting PFK2, ALDOA, ENOα, ENOβ, PDH, ECH1, CPT1A, and R1 affecting PDH, ECH1, HIF, HSP70 (Table 1). This result suggests that although both ASIV and R1 contribute to the energy metabolism improvement potential of QSYQ, the signaling pathway implicated may differ from each other. On the other hand, of DLA-elicited differentially expressed proteins 44% were related to oxidative stress, which, together with the finding that DLA was the only mono-therapy that significantly reduced MDA after AAS2M, demonstrated DLA as the major contributor to antioxidant effect of QSYQ. Furthermore, DLA modulated SODM, RhoGDI1 and HSPB1 in CH (Table 1), suggesting involvement of these proteins in DLA signaling. ASIV and R1 were also found to affect the expression of SODM and HSPB1 (both ASIV and R1), or RhoGDI1 (R1) to some extent. However, either ASIV or R1 mono-therapy did not exert any significant effect on MDA content, suggesting that some other critical links exist that mediate DLA effect, in addition to SODM, RhoGDI1 and HSPB1.

In designing a formula in traditional Chinese medicine, some adjuvant components should be considered to facilitate the delivery of the principal elements to the targets in the body^16^. An interesting finding of the present study was that DO alone did not attenuate CH, nor affected any proteins that were tested for energy metabolism or oxidative stress but with CPT1A as an exception. CPT1A is known to be an enzyme that helps fatty acids cross the inner membrane of mitochondria and thus produce energy inside mitochondria^42^. This result highlights the special role of DO as adjuvant in QSYQ formulation. This notion is also supported by proteomic result showing that DO mainly affected proteins involved in ion transport (40%) and protein modification (40%).

The interaction among different ingredients in a compound medicine has long been recognized in traditional Chinese medicine. This interaction may lead to strengthening or weakening the efficiency relative to any single ingredient, even creating a novel effect. The art of formulating a compound Chinese medicine is to manipulate these interactions to yield a regime with highest efficiency and lowest side effect. The dose of QSYQ used in the present study was its equivalent clinic dose, while the doses of ASIV, DLA and R1 were those that contained in QSYQ used. The content of DO in QSYQ is very low (0.4%). More than 20 different components are so far isolated from DO, none of which is reported having potential to protect against cardiac hypertrophy. The only beneficial role observed for DO in QSYQ is to help absorb other components and potentiate their effects^43^. These facts led us to use DO but not it’s any individual ingredient in the present study. The result of the present study provides an example showing how the interactions among the different ingredients look like. To this end, ASIV+DLA intensified the effect of ASIV on ALDOA and SODM; ASIV+R1 intensified the effect of ASIV on SODM; ASIV+DLA+R1 intensified the effect of ASIV and R1 on ATP/ADP and ATP/AMP, the effect of ASIV on ALDOA, the effect of DLA on ENOβ, the effect of R1 on HIF1, the effect of DLA on MDA and the effect of ASIV and R1 on SODM (Table 1). On the contrary, we found that although ASIV mono-therapy could modulate ECH1, CPT1A and SODM, these effects lost when ASIV combined with other ingredient, for example, ASIV+DLA lost the effect on ECH1 and CPT1A; ASIV+R1 lost the effect on CPT1A; ASIV+DO lost the effect on SODM (Table 1). Interestingly, we found that two or more ingredients combined together exerted some effects that any single one alone did not have, such as DLA and R1 mono-therapy had no effect on CPT1A, while DLA+R1 could modulate CPT1A; ASIV, DLA and DO mono-therapy had no effect on HIF1 and HSP70, while ASIV+DLA and ASIV+DO could modulate these two proteins; ASIV and R1 mono-therapy had no effect on MDA, while ASIV+R1 could modulate MDA (Table 1). More importantly, QSYQ, a combination of all the ingredients, always revealed the highest efficiency regardless of evaluating by variables of CH or energy metabolism or oxidative stress, demonstrating the rationality of QSYQ formula.

Nevertheless, the present study has some limitations. (1) Proteomic analysis revealed that 8 function groups of proteins were differentially expressed after treatments, of which only oxidative stress and energy metabolism were evaluated in detail in present study regarding the relative contribution of each ingredient of QSYQ. However, some other proteins, such as those involved in apoptosis and inflammation, are also known as important determinants in progression of cardiac hypertrophy. The relative contribution of each ingredient of QSYQ to attenuate these insults remains unknown and needs further investigation; (2) QSYQ is composed of 4 herbs, each of which contains a number of active components. In addition to those that were evaluated in the present study, what is the contribution of other ingredients contained in each herbs to the effect of QSYQ remains to be explored.

In conclusion, the compound Chinese medicine QSYQ was able to effectively attenuate pressure overload-induced CH of rat. The efficiency of QSYQ benefits from its multi-components and multi-targets mode, with ASIV and R1 mainly contributing to energy metabolism modulation, DLA to protection of oxidative stress, while DO acting as an adjuvant. This result demonstrates the advantage of traditional Chinese medicine in coping with complex diseases.

## Materials and Methods

### Animals

Male Sprague-Dawley rats, weighing 95–105 g, were purchased from the Animal Center of Peking University Health Science Center. Animals were raised at a temperature of 20 ± 2 with 12/12-h light/dark cycles and fed with standard rat chow and water. Animal care was in compliance with institutional guidelines of the Peking University Animal Research Committee, and experimental protocols were approved by the Committee on the Ethics of Animal Experiments of the Health Science Center of Peking University (LA2013-72).

### Drugs and reagents

QSYQ (Batch number: 20120914) and DO (Batch number: 20121101) were obtained from Tasly Pharmaceutical Co. Ltd. (Tianjin, China). QSYQ was manufactured according to the guidelines of Good Manufacturing Practice and Good Laboratory Practice, and the content of its major components was determined by high performance liquid chromatography. The proportions of Astragalus membranaceus (Huangqi), Salvia miltiorrhiza (Danshen), Panax notoginseng (Sanqi), and Dalbergia odorifera (Jiangxiang, DO) in QSYQ are 60%, 30%, 6%, 0.4%, respectively, with the content of ASIV in Astragalus membranaceus being 1.1 mg/g, DLA in Salvia miltiorrhiza being 2.42 mg/g, and R1 in Panax notoginseng being 10 mg/g. QSYQ and DO were dissolved in saline (Beijing Chemical Works, Beijing, China) to make a solution at concentration of 0.2 g/mL^21^,^44^ and 0.8 mg/mL, respectively. ASIV, DLA and R1 (purity ≥ 99.9%) were obtained from Fengshanjian Medicine Research Co. Ltd. (Kunming, Yunnan, China), and dissolved in saline to make a solution at concentration of 0.132 mg/mL, 0.848 mg/mL and 0.12 mg/mL, respectively.

Pentobarbital sodium was purchased from Genview (St. Galveston, TX, USA). Isoflurane was from Jiupai Pharmaceutical Co. Ltd. of HeBei (Jiu-pai, Shijiazhuang, China). HE was from Zhongshan Goldenbridge biotechnology Co. Ltd (Beijing, China). Wheat germ agglutinin (WGA) and rhodamine phalloidine were purchased from Invitrogen (Carlsbad, CA, USA). Tris, urea, 3-[(3-cholamidopropyl) dimethylammonio]-2-hydroxy-1-propanesulfonate (CHAPS), DL-Dithiothreitol (DTT), sodium dodecyl sulfate (SDS) and iodoacetamide were purchased from AMRESCO (Solon, OH, USA). Glycerol and paraformaldehyde were from Beijing Chemical Works (Beijing, China). Thiourea and PhastGel Blue R were purchased from GE Healthcare (Piscataway, NJ, USA). Trypsin solution was purchased from Sigma-Aldrich (St. Louis, MO, USA). The antibodies against reduced glyceraldehyde-phosphate dehydrogenase (GAPDH), ALDOA, ENOβ, ECH1, HSP70, HIF1, CPT1A, PFK2, HSPB1 and RhoGDI1 were from Abcam (Cambridge, USA), the antibodies against ENOα, SODM and PDH were from Santa Cruz Biotechnology (Santa Cruz, Calif, USA). ELISA kits for ATP, ADP, AMP, and MDA were purchased from Beijing Huanya Biomedicine Technology Co. Ltd (Beijing, China).

### Surgical protocols

The animals were fasted for 12 hours before experiment, while allowing free access to water. Animals were anesthetized with 2% pentobarbital sodium (60 mg/kg) by intraperitoneal injection. After tracheotomy, the animals were ventilated with a positive pressure respirator (ALC-V8, Shanghai, China). The thorax was opened and ascending aortic stenosis was implemented by placing a silver clip (0.9-mm inside diameter) on the ascending aorta. Sham-operated animals underwent an identical procedure but without the clip^12^.

### Experimental groups

After 1 month (30 days) of AAS, the animals were examined by echocardiography, and the rats having left ventricular wall thickness 20% thicker than that in sham group were identified as succeeded in modeling and randomly assigned either to model groups, including AAS1M and AAS2M, or drug treatment groups, including QSYQ, ASIV, DLA, R1, DO, ASIV+DLA, ASIV+R1, ASIV+DO, DLA+R1, DLA+R1+DO, and ASIV+DLA+R1 group, 6 animals in each group. The animals in AAS1M were sacrificed for assessment of parameters concerned after echocardiographic analysis. In Sham and AAS2M groups, the animals received saline daily by gavage for the succeeding 1 month at a dose of 4 mL/kg/day. Over the same period of time, the animals in drug treatment groups received indicated drugs by gavage at following doses: 0.8 g/kg/day (QSYQ), 0.528 mg/kg/day (ASIV), 3.392 mg/kg/day (DLA), 0.48 mg/kg/day (R1), and 3.2 mg/kg/day (DO). Two months after AAS, the animals were examined by echocardiography once again and then sacrificed for parameter assessment.

### Echocardiographic analysis

The left ventricle function was evaluated 1 and 2 months after AAS, respectively, using a Vevo 770 High-Resolution Imaging Systems (Visual Sonics Inc, Toronto, ON, Canada) with a 17.5 MHz linear array transducer. Briefly, rats (n = 6) were anaesthetized with 1.5–2.0% isoflurane. Two-dimensional cine loops and guided M-mode frames were recorded from the parasternal short and long axis^12^. The following parameters were measured as indicators of cardiac function or remodeling: LVPWd, LVPWs, LVIDs, EF and FS.

### MBF

Following echocardiographic analysis two months after AAS, animals (n = 6) underwent thoracotomy, and MBF was measured by Laser-Doppler Perfusion Imager (PeriScan PIM3, Perimed, Sweden) equipped with a computer. All images were evaluated with the software LDPIwin 3.1 (Perimed, Sweden)^40^. For each animal, MBF was the average of three independent measurements.

### HW/BW and histology

Rats (n = 6) were sacrificed after MBF evaluation, and the hearts were perfused with saline and then collected. Both BW and HW were determined, and HW/BW was calculated to evaluate the hypertrophic response to pressure over-load.

The obtained hearts were fixed in 4% paraformaldehyde solution for 48 hours, and processed for paraffin section (5 μm). Sections were stained with HE, WGA and rhodamine phalloidine. The sections were observed with microscope (BX512DP70, Olympus, Tokyo, Japan) or laser scanning confocal microscope (TCS SP5, Leica, Mannheim, Germany), and five fields were randomly selected for evaluation. Cardiomyocyte cross section area was determined on sections stained with WGA in randomly selected 5 fields, calculating the average of cross section areas of 3–5 cells in each, using Image-Pro Plus 6.0 (Media Cybernetic, Bethesda, MD, USA).

### Proteome analysis

For proteome analysis, two-dimensional polyacrylamide gel electrophoresis was performed. Briefly, the heart tissue (n = 3) was homogenized in a homogenization buffer containing 30 mM Tris, 7 M urea, 2 M thiourea, 4% CHAPS, and 40 mM DTT. The lysates obtained were centrifuged at 15, 294 *g* for 30 minutes at 4 °C; the supernatant was collected and stored as aliquots at −80 °C until use. The samples were pre-treated with 2D Clean-Up Kit (GE Healthcare, Piscataway, NJ, USA) to remove the interfering components. The protein concentration of each sample was determined using the Bradford method. A total of 1 mg of protein was loaded onto a 24-cm linear IPG strip (pH 3–10, Amersham Biosciences, Piscataway, NJ, USA) for first-dimension isoelectric focusing. The strips were placed into a Ettan IPGphor 3 (GE Healthcare, Piscataway, NJ, USA) and rehydrated at 50 V for 12 hours, and the proteins were separated based on their protein isoelectric point with a protocol as follows: 250 V with linear climb for 30 minutes, 500 V with linear climb for 30 minutes, 1000 V with linear climb for 60 minutes, 4000 V with rapid climb for 60 minutes, 8000 V with linear climb for 5 hours, and 8000 V with rapid climb until a level of 60000 V-h was reached. After first-dimension isoelectric focusing, the IPG strips were equilibrated for 15 minutes in a buffer containing 50 mM Tris-HCl, pH 8.8, 30% glycerol, 7 M urea, 2% SDS, and 1% DTT, then further treated in a similar buffer containing 4% iodoacetamide instead of DTT for 15 minutes and directly applied onto 12% homogeneous SDS-PAGE gels for 6 hours electrophoresis using a Ettan DALT six system (GE Healthcare, Piscataway, NJ, USA). The gels were stained by PhastGel Blue R according to the manufacturer’s instructions. The stained gels were scanned and then analyzed using Image Master 2D platinum 7 software (GE Healthcare, Piscataway, NJ, USA). The intensity of each spot was quantified by calculating the spot volume after the gel image had been normalized. Each sample was processed in triplicate to ensure reproducibility, and the Student’s t-test was used to evaluate the average change in spot abundance corresponding to each target spot across the gels. Spots with more than a 2-fold change (either increasing or decreasing) in density were considered to be differentially expressed (paired t-test, p ≤ 0.05) and were selected for further identification via mass spectrometry analysis^9^. Briefly, differently expressed spots were manually excised from the gels, destained and dehydrated in acetonitrile. Subsequently, they were subjected to rehydration with a trypsin solution and in-gel digestion overnight at 37 °C. For each spot, 0.75 μL of digested peptides were spotted onto a MALDI-TOF target (GE Healthcare, Piscataway, NJ, USA) and allowed to dry. Then, 0.75 μL of matrix solution (saturated solution of α-cyano-4-hydroxycinnamic acid in 50% v/v acetonitrile and 0.5% v/v trifluoroacetic acid) was applied to the dried sample, and tryptic peptide masses were acquired on an Ettan MALDI-ToF Pro mass spectrometer (GE Healthcare, Piscataway, NJ, USA). Mass spectra were acquired in reflectron mode with an accelerating voltage of 20 kV using the Ettan MALDI Evaluation software (GE Healthcare, Piscataway, NJ, USA). Peptide masses were internally calibrated using the 842.509 and 2211.105 m/z trypsin autolysis peptides. Databases were searched for peptide identification using MASCOT Distiller 2.1 (Matrix Science, http://www.matrixscience.com).

MetaCore (GeneGo, St Joseph, MI) was used to and map the differently expressed proteins into biological networks and the integrated software was applied for functional interpretation of the protein data. MetaCore is based on a manually curated database of human protein-protein interactions, protein-DNA interactions, transcriptional factors, metabolic, and signaling pathways. Differentially expressed proteins were converted into gene symbols and uploaded into MetaCore for analysis. For network analysis, the shortest paths algorithm was used to deduce top scoring processes that are regulated by differentially expressed proteins^45^.

### Western Blotting Assay

A piece of about 200 mg of myocardium tissue was harvested from each animal (n = 4), quickly frozen in liquid nitrogen, and stored at −80 °C for a maximum of one week before use. The whole protein of the tissues was extracted with a protein extraction kit (Applygen Technologies, Beijing, China), according to manufacturer’s instruction. The whole protein was separated on 12% SDS-PAGE and transferred to polyvinylidene difluoride membrane. The membrane was incubated overnight at 4 °C with antibodies, respectively, against GAPDH (1:5000), ALDOA (1:5000), ENOα (1:4000), ENOβ (1:4000), ECH1 (1:1000), HSP70 (1:1000), HIF1 (1:500), CPT1A (1:2000), PFK2 (1:4000), PDH (1:4000), SODM (1:8000), HSPB1 (1:800) and RhoGDI1 (1:4000). The membranes were then incubated with secondary antibody for one hour at room temperature, and immunoreactive bands were revealed using an enhanced chemiluminescence system. The protein signal in the X-film was quantified by scanning densitometry and further evaluated by a bio-image analysis system (Quantity One image analyzer software, Bio-Rad, Richmond, CA, USA).

### ELISA Assay

The whole protein was extracted as described above (n = 6). The content of ATP, ADP, AMP and MDA in myocardium was assessed with ELISA by microplate reader (MULTISKAN MK3, Thermo, Minnesota, USA), according to the manufacturer’s instructions. The assay sensitivities of ATP, ADP, AMP and MDA ELISA kits are 3.3 ng/mL–200 ng/mL; 30 nmol/L–1000 nmol/L; 30 nmol/L–1100 nmol/L and 0.1 nmol/mL, respectively. The assay was performed in triplicate for each sample.

### Real-Time PCR

Real-time quantitative PCR was performed to detect the mRNA level of ANP, BNP and MYH-7 from each sample (n = 6). For this, total RNA was isolated from rat hearts using TRIZOL reagent (Carlsbad, CA, USA), and the RNA was applied for reverse transcription using a Revert Aid First Strand cDNA Synthesis Kit (Fermentas Lifesciences, Vilnius, Lithuania) to generate the first strand cDNA mix. Real-time PCR was performed utilizing the ABI PRISM sequence detection system 7500 (Perkin-Elmer Applied Biosystems, CA, USA). Primer concentrations were 10 μmol/L. Primer sequences (all Rattus) used were as follows: ANP-forward, 5′-GAG GAG AAG ATG CCG GTA G-3′, ANP-reverse, 5′-CTA GAG AGG GAG CTA AGT G-3′; BNP-forward, 5′-TGA TTC TGC TCC TGC TTT TC-3′, BNP-reverse, 5′-GTG GAT TGT TCT GGA GAC TG -3′; MYH-7-forward, 5′-TTG GCA CGG ACT GCG TCA TC-3′, MYH-7-reverse, 5′-GAG CCT CCA GAG TTT GCT GAA GGA-3′; GAPDH-forward, 5′-ATG CTG GTG CTG AGT ATG TC-3′, GAPDH-reverse, 5′-AGT TGT CAT ATT TCT CGT GG-3′. The PCR reaction mixture (25 μL) included 2x Maxima SYBR Green/ROX qPCR Master Mix, reverse transcription product cDNA, forward and reverse primers, nuclease-free water. The reactions took place in a 96-well plate at 50 °C for two minutes, 95 °C for 10 minutes, followed by 40 cycles of 95 °C for 15 seconds, 58 °C for one minute and plate read. A melting curve was used to check the specificity of an amplified product. All tests were performed in triplicate. All data were quantified by use of the comparative CT method.

### Statistical analysis

All parameters were expressed as mean ± SE. Statistical analysis was carried out with SAS 9.3 statistical software (SAS Institute Inc., Carry, NC, USA), and one-way analysis of variance was used, then for post hoc testing, Turkey test was used for multiple comparisons between groups. A probability of less than 0.05 was considered to be statistically significant.

## Additional Information

**How to cite this article**: Chen, Y.-Y. *et al.* QiShenYiQi Pills, a compound in Chinese medicine, protects against pressure overload-induced cardiac hypertrophy through a multi-component and multi-target mode. *Sci. Rep.* **5**, 11802; doi: 10.1038/srep11802 (2015).

## Supplementary Material

Supplementary Information

## Figures and Tables

**Table 1:** The summary of the effect and efficient comparison of each drug treatment.

	**ATP/ADP**	**ATP/AMP**	**PFK2**	**ALDOA**	**ENOα**	**ENOβ**	**PDH**	**ECH1**	**CPT1A**	**HIF1**	**HSP70**	**MDA**	**SODM**	**RhoGDI1**	**HSPB1**
QSYQ	†$€	†$€	†	†$	†$§	†$§	†	†	†	†€	†	†§	†$§€	†§€	†
ASIV	†	†	†	†	†	†	†	†	†				†		†
DLA					†	†	†					†	†	†	†
R1	†	†					†	†		†	†		†	†	†
DO									†						
ASIV + DLA	†	†	†	†$	†	†	†	↓	↓$	↑$§	↑$§	†	†$	†	†
ASIV + R1	†	†	†	†	†	†	†	†	↓$	†	†	↑$€	†$	†	†
ASIV + DO	†	†	†	†	†	†	†	†	†	↑$Ψ	↑$Ψ		↓$		†
DLA + R1	†	†			†	†	†	†	↑§€	†	†	†	†	†	†
DLA + R1 + DO	†	†			†	†	†	†	†	†	†	†	†	†	†
ASIV + DLA + R1	†$€	†$€	†	†$	†	†§	†	†	†	†€	†	†§	†$€	†	†

^†^p < 0.05 vs. AAS2M, ^$^p < 0.05 vs. ASIV, ^§^p < 0.05 vs. DLA, ^€^p < 0.05 vs. R1. ↓: abolish. ↑: create.
