# Supplementary material for: QiShenYiQi Pills, a compound in Chinese medicine, protects against pressure overload-induced cardiac hypertrophy through a multi-component and multi-target mode
Source: Sci Rep. 2015 Jul 2;5:11802. doi: 10.1038/srep11802 (PMC4488877; doi:10.1038/srep11802)
Supplement: Supplementary Information [file srep11802-s1.doc]

**QiShenYiQi Pills, a compound in Chinese medicine, protects against pressure overload-induced cardiac hypertrophy through a multi-component and multi-target mode**

Yuan-Yuan Chen, Quan Li, Chun-Shui Pan, Li Yan, Jing-Yu Fan, Ke He, Kai Sun, Yu-Ying Liu, Qing-Fang Chen, Yan Bai, Chuan-She Wang, Bing He, Ai-Ping Lv, Jing-Yan Han

**Supplementary Materials:**

Table S1

Figures S5a-S7a

Table S1. The results of 54 spots by mass spectrometry analysis and biological functional classification

| Spot Number | Accession | Gene ID | Protein Definition | Molecular Weight (kDa) | Score | Sequence coverage (%) | Biological functions |
| --- | --- | --- | --- | --- | --- | --- | --- |
| 1 | PTGR2_RAT | Q5BK81 | Prostaglandin reductase 2 | 39 | 83 | 10 | oxidative stress |
| 3 | ALDR_RAT | P07943 | Aldose reductase | 36 | 819 | 31 | oxidative stress |
| 4 | ALDR_RAT | P07943 | Aldose reductase | 36 | 819 | 22 | oxidative stress |
| 5 | COQ7_RAT | Q63619 | Ubiquinone biosynthesis protein COQ7 homolog (Fragment) | 20 | 427 | 21 | oxidative stress |
| 11 | SODM_RAT | P07895 | Superoxide dismutase [Mn], mitochondrial | 25 | 964 | 23 | oxidative stress |
| 13 | ESTD_RAT | B0BNE5 | S-formylglutathione hydrolase | 32 | 618 | 22 | oxidative stress |
| 17 | PDIA3_RAT | P11598 | Protein disulfide-isomerase A3 | 57 | 263 | 22 | oxidative stress |
| 23 | COQ9_RAT | Q68FT1 | Ubiquinone biosynthesis protein COQ9, mitochondrial | 35 | 620 | 20 | oxidative stress |
| 33 | CRYM_RAT | Q9QYU4 | Mu-crystallin homolog | 34 | 241 | 17 | oxidative stress |
| 36 | GPX1_RAT | P04041 | Glutathione peroxidase 1 | 22 | 275 | 12 | oxidative stress |
| 41 | ETFA_RAT | P13803 | Electron transfer flavoprotein subunit beta | 28 | 683 | 21 | oxidative stress |
| 2 | D3ZZN3_RAT | D3ZZN3 | Protein Acss1 | 22 | 2451 | 64 | energy metabolism |
| 7 | ATPA_RAT | P15999 | ATP synthase subunit alpha, mitochondrial | 60 | 1910 | 40 | energy metabolism |
| 8 | ATPA_RAT | P15999 | ATP synthase subunit alpha, mitochondrial | 60 | 1940 | 55 | energy metabolism |
| 9 | ATPA_RAT | P15999 | ATP synthase subunit alpha, mitochondrial | 60 | 1910 | 56 | energy metabolism |
| 10 | D4A0T0_RAT | D4A0T0 | Protein Ndufb10 | 21 | 1593 | 73 | energy metabolism |
| 12 | NDUAA_RAT | Q561S0 | NADH dehydrogenase [ubiquinone] 1 alpha subcomplex subunit 10, mitochondrial | 41 | 1330 | 36 | energy metabolism |
| 20 | NDUAA_RAT | Q561S0 | NADH dehydrogenase [ubiquinone] 1 alpha subcomplex subunit 10, mitochondrial | 41 | 688 | 26 | energy metabolism |
| 14 | ECH1_RAT | Q62651 | Delta(3,5)-Delta(2,4)-dienoyl-CoA isomerase, mitochondrial | 36 | 682 | 23 | energy metabolism |
| 15 | ECH1_RAT | Q62651 | Delta(3,5)-Delta(2,4)-dienoyl-CoA isomerase, mitochondrial | 36 | 682 | 26 | energy metabolism |
| 26 | PEBP1_RAT | P31044 | Phosphatidylethanolamine-binding protein 1 | 21 | 698 | 23 | energy metabolism |
| 35 | HPRT_RAT | P27605 | Hypoxanthine-guanine phosphoribosyltransferase | 25 | 516 | 21 | energy metabolism |
| 44 | ALDOA_RAT | P05065 | Fructose-bisphosphate aldolase A | 40 | 1600 | 46 | energy metabolism |
| 45 | IDH3B_RAT | Q68FX0 | Isocitrate dehydrogenase [NAD] subunit beta, mitochondrial | 43 | 1030 | 38 | energy metabolism |
| 46 | ODPA_RAT | P26284 | Pyruvate dehydrogenase E1 component subunit alpha, somatic form, mitochondrial | 44 | 1160 | 56 | energy metabolism |
| 47 | ODPA_RAT | P26284 | Pyruvate dehydrogenase E1 component subunit alpha, somatic form, mitochondrial | 44 | 1240 | 47 | energy metabolism |
| 48 | ACOT2_RAT | O55171 | Acyl-coenzyme A thioesterase 2, mitochondrial | 50 | 751 | 30 | energy metabolism |
| 49 | ACADS_RAT | P15651 | Short-chain specific acyl-CoA dehydrogenase, mitochondrial | 45 | 1770 | 49 | energy metabolism |
| 50 | ALDH2_RAT | P11884 | ATP synthase subunit alpha, mitochondrial | 57 | 115 | 8 | energy metabolism |
| 54 | ENOB_RAT | P15429 | Beta-enolase | 47 | 1340 | 45 | energy metabolism |
| 52 | GUAD_RAT | Q9WTT6 | Guanine deaminase | 52 | 546 | 26 | energy metabolism |
| 6 | HSPB6_RAT | P97541 | Chaperone; Heat shock protein beta-6 | 18 | 300 | 13 | chaperon |
| 25 | HSPB1_RAT | P42930 | Heat shock protein beta-1 | 23 | 313 | 12 | chaperon |
| 28 | HSPB1_RAT | P42930 | Heat shock protein beta-1 | 23 | 707 | 27 | chaperon |
| 30 | HSPB1_RAT | P42930 | Heat shock protein beta-1 | 23 | 432 | 19 | chaperon |
| 27 | HSPB2_RAT | O35878 | Heat shock protein beta-2 | 20 | 293 | 14 | chaperon |
| 32 | HSPB7_RAT | Q9QUK5 | Heat shock protein beta-7 (Fragment) | 10 | 261 | 10 | chaperon |
| 37 | CRYAB_RAT | P23928 | Alpha-crystallin B chain | 20 | 157 | 12 | chaperon |
| 38 | CRYAB_RAT | P23928 | Alpha-crystallin B chain | 20 | 469 | 20 | chaperon |
| 39 | CRYAB_RAT | P23928 | Alpha-crystallin B chain | 20 | 547 | 22 | chaperon |
| 40 | CRYAB_RAT | P23928 | Alpha-crystallin B chain | 20 | 424 | 17 | chaperon |
| 53 | HSP70_RAT | Q07439 | Heat shock 70 kDa protein 1A/1B | 70 | 383 | 27 | chaperon |
| 18 | ARHL1_RAT | Q5XIB3 | [Protein ADP-ribosylarginine] hydrolase-like protein 1 | 40 | 531 | 29 | protein modification |
| 19 | ARHL1_RAT | Q5XIB3 | [Protein ADP-ribosylarginine] hydrolase-like protein 1 | 40 | 499 | 21 | protein modification |
| 51 | FIBG_RAT | P02680 | Fibrinogen gamma chain | 51 | 418 | 22 | inflammatory response |
| 16 | VDAC1_RAT | Q9Z2L0 | Voltage-dependent anion-selective channel protein 1 | 31 | 1460 | 26 | ion transport |
| 21 | TCTP_RAT | P63029 | Translationally-controlled tumor protein | 20 | 217 | 14 | ion transport |
| 29 | CLIC4_RAT | Q9Z0W7 | Chloride intracellular channel protein 4 | 29 | 623 | 22 | ion transport |
| 42 | VDAC2_RAT | P81155 | Voltage-dependent anion-selective channel protein 2 | 32 | 495 | 19 | ion transport |
| 24 | GDIR1_RAT | Q5XI73 | Rho GDP-dissociation inhibitor 1 | 23 | 435 | 17 | apoptosis |
| 31 | FRIH_RAT | P19132 | Ferritin heavy chain | 21 | 313 | 16 | apoptosis |
| 34 | FHL2_RAT | O35115 | Four and a half LIM domains protein 2 | 34 | 879 | 29 | apoptosis |
| 22 | ANXA5_RAT | P14668 | Annexin A5 | 36 | 1360 | 51 | blood coagulation |

1. Spot numbers match those present in Figure 4a. 2. Gene ID used in MetaCore analysis is given next to the each accession. 3. Probability-based molecular weight search score was obtained during peptide matching using MASCOT (http://www.matrixscience.com).

**Supplementary Figures S5a-S7a**

Figure S5a


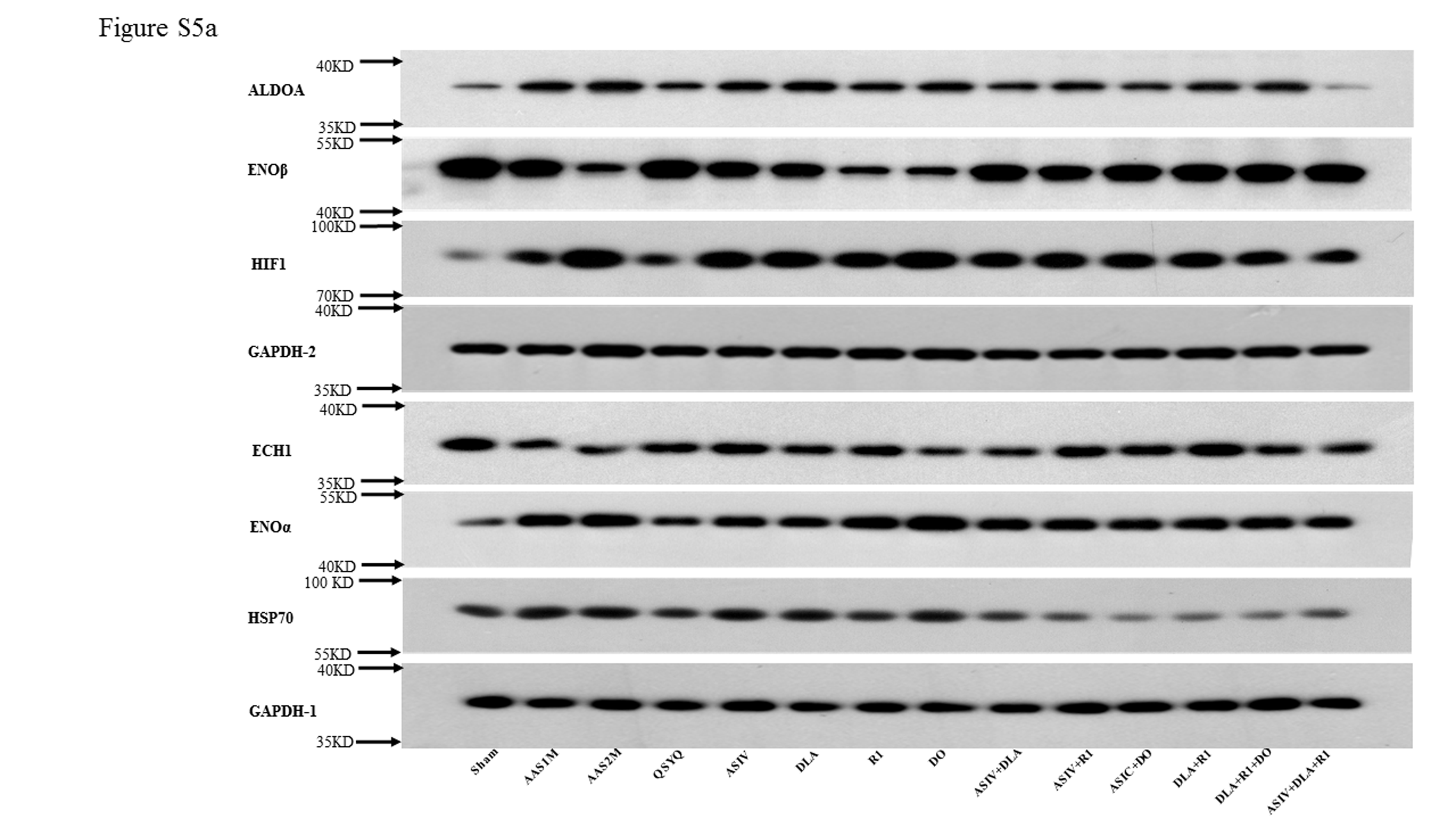


**Figure S5a.** The representative western blotting bands of each protein in different groups. The bands of ALDOA, ENOβ, HIF1 and GAPDH-2 were cropped from one gel, while the bands of ECH1, ENOα, HSP70 and GAPDH-1 were cropped from another gel, as shown with indication of molecular size.

Figure S6a


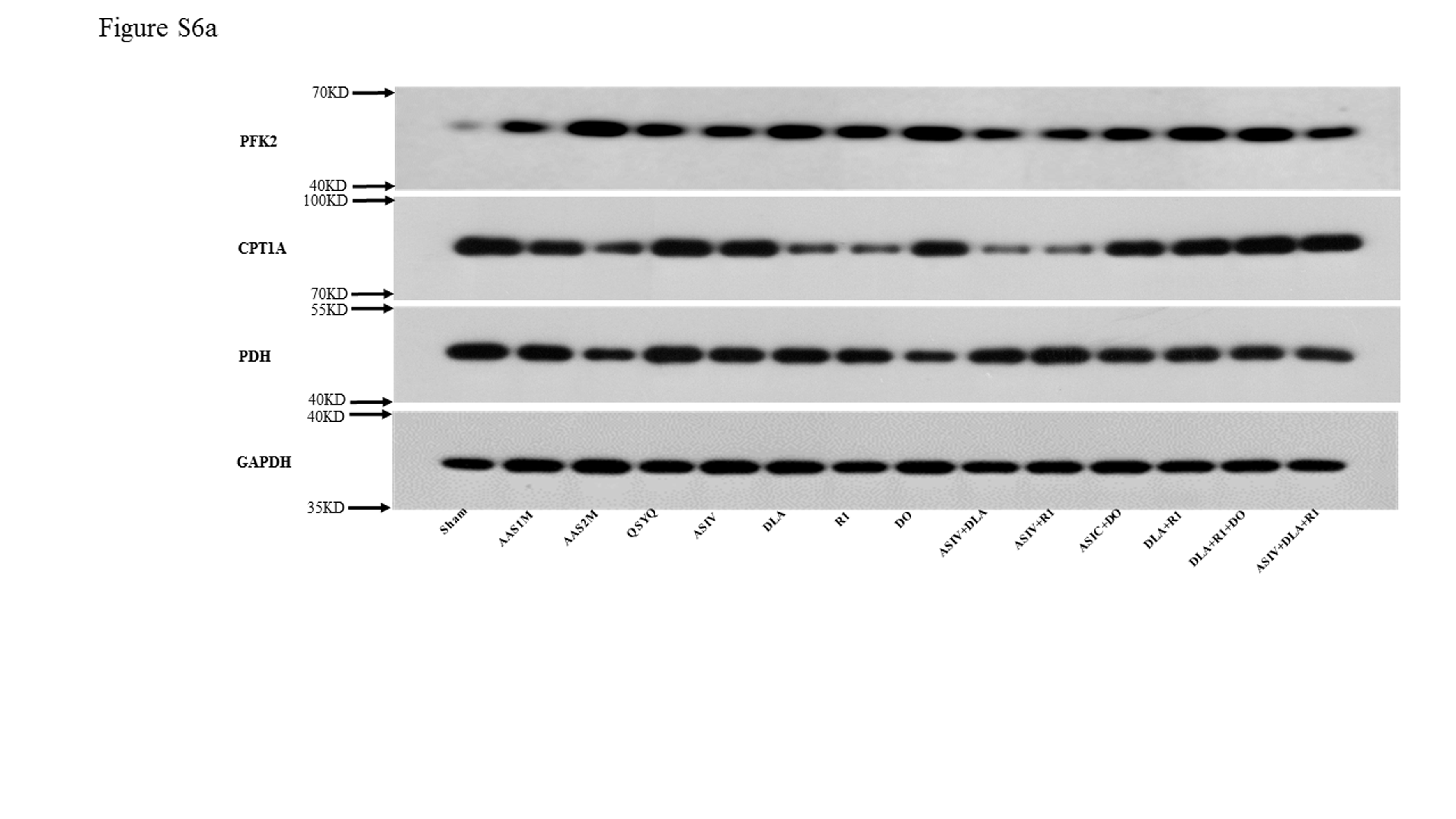


**Figure S6a.** The representative western blotting bands of PFK2, CPT1A and PDH in different groups. All bands in figure 6 were cropped from one gel, as shown with indication of molecular size.

Figure S7a


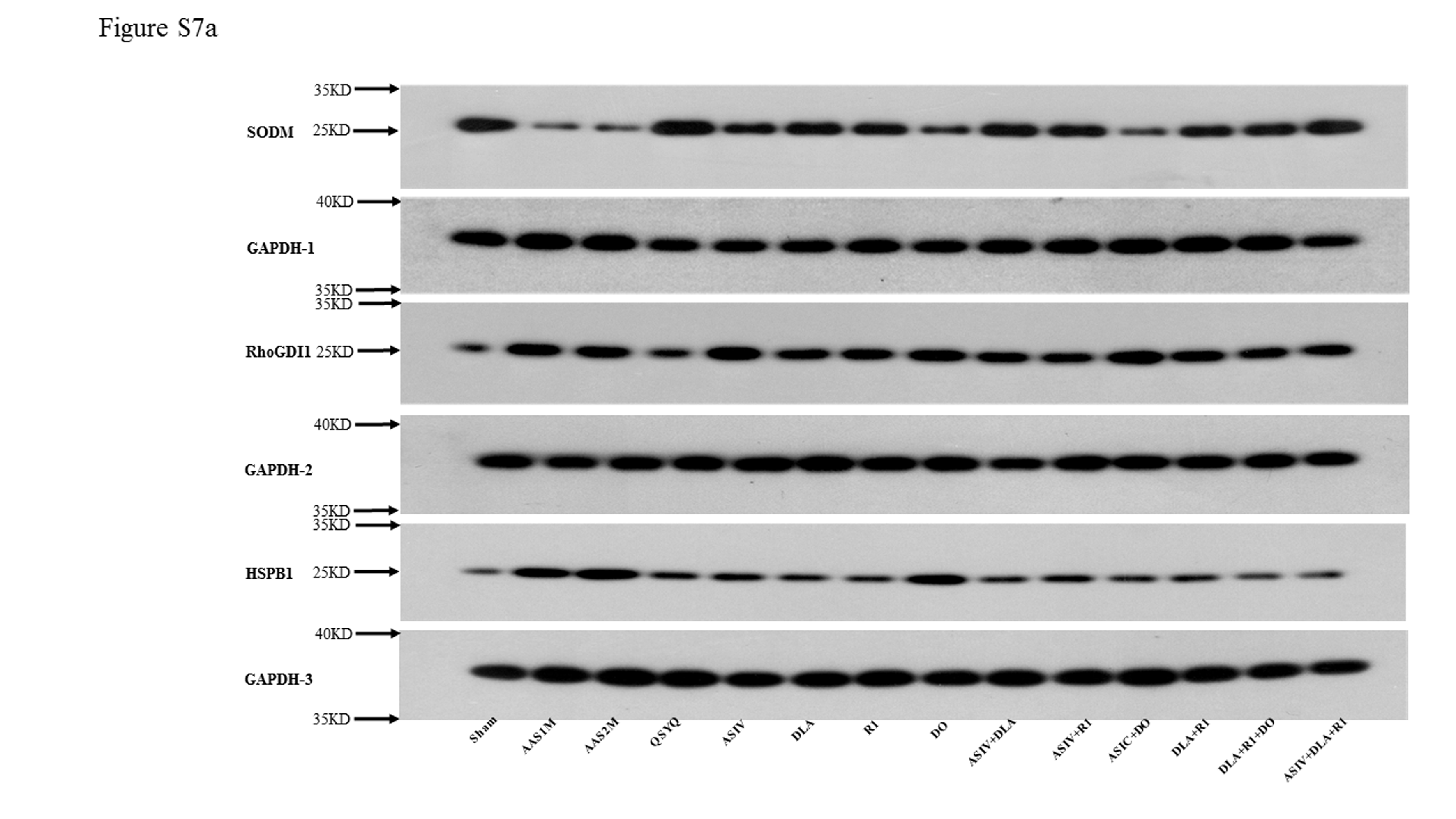


**Figure S7a**. The representative western blotting bands of SODM, RhoGDI1 and HSPB1 in different groups. The bands of SODM and GAPDH-1 were cropped from one gel, and the bands of RhoGDI1 and GAPDH-2 were cropped from one gel, and the bands of HSPB1 and GAPDH-3 were cropped from one gel, as shown with indication of molecular size.
